# Comment on “High Infertility Rates and Pregnancy Complications in Female Physicians Indicate a Need for Cultural Change”

**DOI:** 10.1097/AS9.0000000000000265

**Published:** 2023-03-01

**Authors:** Spandana Jagannath, Andrew G. Hill, Sabaretnam Mayilvaganan

**Affiliations:** From the * Department of Endocrine and Breast Surgery, Sanjay Gandhi Postgraduate Institute of Medical Sciences, India; † University of Auckland, Middlemore Hospital, Auckland, New Zealand

We read with interest the article “High infertility rates and pregnancy complications in female physicians indicate a need for cultural change” by Lai et al.^1^ We congratulate the authors on shedding light on this important issue in female physicians.

Particularly early in their careers, female physicians struggle to balance the demands of work and family, having to fulfill the roles of a responsible physician, a loving spouse, a caring daughter, and a dedicated mother to the family. In this scenario, the role of multitasking can take its toll and lead to career burnout.

Female doctors have historically postponed conception until after they finished their training, despite known obstetric and infertility concerns after the age of 35.^2–4^ In Low and Medium countries, like India, medical education is expensive and financial constraints and inconsistent policies regarding maternity leave can also be a reason for delaying pregnancy.

Surgical education is also not uniform throughout the world. In India, it is at least a 10-year long process of tough competitive entrance and exit exams, and vigorous academic and practical training. To reach the touchline, female physicians have to strive hard in their prime childbearing years and, if not carefully planned, this is a real struggle, both on personal and professional fronts.

During the child’s formative years, raising them alone can present a challenge that extended family can help resolve, a tradition, still prevalent in some Asian countries. However, this may be detrimental to a close mother-child relationship while burdening aging grandparents, ultimately leading to career dissatisfaction.

In this context, changing surgical culture to support pregnancy is paramount to foster and encourage female physicians during their early career stages as has been recommended by many studies.^5,6^

We have few queries that we would appreciate comment on:

1. General surgery is seen as having an extremely tough training programme, perhaps harder than other surgical specialities. Did the authors consider that including all surgical specialities may have resulted in bias since few subspecialties require as onerous training as that required for general surgery?
2. You used social media for recruitment. Would a more targeted approach using personal email or mail for such a sensitive issue would have resulted in more female physicians participating?
3. Do the authors think that having a society for female physicians would be helpful?

Thank you for your comments on these issues.
